# Resveratrol Attenuates Formaldehyde Induced Hyperphosphorylation of Tau Protein and Cytotoxicity in N2a Cells

**DOI:** 10.3389/fnins.2016.00598

**Published:** 2017-01-31

**Authors:** Xiaping He, Zhenhui Li, Joshua D. Rizak, Shihao Wu, Zhengbo Wang, Rongqiao He, Min Su, Dongdong Qin, Jingkun Wang, Xintian Hu

**Affiliations:** ^1^Key Laboratory of Animal Models and Human Disease Mechanisms of the Chinese Academy of Sciences and Yunnan Province, Kunming Institute of Zoology, Chinese Academy of SciencesKunming, China; ^2^Nerve System Coding Discipline Group, Kunming College of Life Science, University of the Chinese Academy of SciencesKunming, China; ^3^Yunnan Province Company Key Laboratory for TCM and Ethnic Drug of New Drug Creation, Yunnan Bai Yao Group Innovation and R&D Center, Yunnan Institute of Materia MedicaKunming, China; ^4^State Key Laboratory of Brain and Cognitive Science, Institute of Biophysics, Chinese Academy of SciencesBeijing, China; ^5^Key Laboratory of Mental Health, Institute of Psychology, Chinese Academy of SciencesBeijing, China; ^6^CAS Center for Excellence in Brain Science and Intelligence Technology, Chinese Academy of SciencesShanghai, China; ^7^Kunming Primate Research Center, Kunming Institute of Zoology, Chinese Academy of SciencesKunming, China

**Keywords:** Alzheimer's disease, formaldehyde, resveratrol, tau protein, GSK-3β, CaMKII, PP2A

## Abstract

Recent studies have demonstrated that formaldehyde (FA)—induced neurotoxicity is important in the pathogenesis of Alzheimer's disease (AD). Elevated levels of FA have been associated with memory impairments and the main hallmarks of AD pathology, including β-amyloid plaques, tau protein hyperphosphorylation, and neuronal loss. Resveratrol (Res), as a polyphenol anti-oxidant, has been considered to have therapeutic potential for the treatment of AD. However, it has not been elucidated whether Res can exert its neuroprotective effects against FA-induced neuronal damages related to AD pathology. To answer this question, the effects of Res were investigated on Neuro-2a (N2a) cells prior to and after FA exposure. The experiments found that pre-treatment with Res significantly decreased FA-induced cytotoxicity, reduced cell apoptosis rates, and inhibited the hyperphosphorylation of tau protein at Thr181 in a dose-dependent manner. Further tests revealed that this effect was associated with the suppression of glycogen synthase kinase (GSK-3β) and calmodulin-dependent protein kinase II (CaMKII) activities, both of which are important kinases for tau protein hyperphosphorylation. In addition, Res was found to increase the activity of phosphoseryl/phosphothreonyl protein phosphatase-2A (PP2A). In summary, these findings provide evidence that Res protects N2a cells from FA-induced damages and suggests that inhibition of GSK-3β and CaMKII and the activation of PP2A by Res protect against the hyperphosphorylation and/or mediates the dephosphorylation of tau protein, respectively. These possible mechanisms underlying the neuroprotective effects of Res against FA-induced damages provide another perspective on AD treatment via inhibition of tau protein hyperhosphorylation.

## Introduction

Formaldehyde (FA), a highly reactive single carbon aldehyde with the formula CH_2_O or HCHO, is widely distributed in living organisms and environments He et al. (2010). Exposure to FA is known to cause acute health problems, such as upper respiratory illnesses, allergies, and possible death (Tang et al., 2009). More recently, studies have focused on evaluating the association between chronic exposure to FA and its neurotoxicity. An increasing number of reports have revealed that excessive exposure to FA can induce amyloid aggregation (Chen et al., 2007; Rizak et al., 2014) and tau protein aggregation and hyperphosphorylation *in vitro* and *in vivo* (Nie et al., 2007; Lu et al., 2013). Rodent studies have shown that elevation of endogenous FA can lead to memory impairments, tau protein hyperphosphorylation and neuronal loss (Tong et al., 2011, 2013; Yang et al., 2014a). In our previous study with monkeys, the neurotoxicity of FA, induced with the chronic exposure to methanol, was also observed to form β -amyloid plaques and cause memory impairments (Yang et al., 2014b). All of these findings suggest that FA toxicity is related to the main hallmarks of AD pathology. Moreover, data from clinical studies has also found elevation of endogenous FA in urine from dementia patients to be inversely correlated with cognitive impairments (Tong et al., 2011). Similar elevated FA levels have also been observed post-mortem in the hippocampus of AD patients (Tong et al., 2011). This evidence collectively indicates that FA is closely linked to AD pathology, which opens a whole new avenue for Alzheimer's research and drug development.

Resveratrol (Res), as a polyphenol anti-oxidant, has attracted wide attention for its potential as a therapeutic agent in preventing and treating AD (Tredici et al., 1999; Ranney and Petro, 2009). It has been reported that Res at 10–100 μM can exert neuroprotective effects (Richard et al., 2011). *In vivo* and *in vitro* experiments have shown that Res exerted its neuroprotective effects on AD pathological markers through a number of mechanisms, such as by promoting clearance of abnormal Aβ peptides and the anti-amyloidogenic cleavage of β-amyloid precursor protein (APP), as well as by reducing oxidative stress (Marambaud et al., 2005; Huang et al., 2011). Interestingly, Res has also been identified as a natural formaldehyde capturer (Tyihák et al., 1998). Together, the above-mentioned evidence suggests that the neuroprotective effects of Res have potential to reduce AD pathology. However, it is not clear whether Res can protect neurons from FA-induced damages.

To answer this question, the protective effects of Res were investigated in FA-treated N2a cells, a mouse neuroblastoma cell line widely used to study neurotoxicity (LePage et al., 2005). Then, the underlying mechanisms of this protection were further explored.

## Materials and methods

### Reagents

Antibodies and dyes used for western blotting and/or immunostaining were obtained from the following resources: Anti-β-actin (ab6276), anti-PP2A (ab32141), anti-CaMKII (ab52476), anti-GSK3β (Y216) (ab85305), Goat anti-Mouse/ Donkey anti-Rabbit IgG horseradish peroxidase (HRP)-conjugated secondary antibodies (ab6789)/(ab6802) were purchased from Abcam (Cambridge, UK); anti-total tau [Tau5, a monoclonal antibody recognizing both phosphorylated and non-phosphorylated tau (MAB361)] was purchased from Merck Millipore (Massachusetts, U.S.A.); anti-phosphorylated tau at Thr181 (pT181) (SAB11107) was purchased from Signalway Antibody (Maryland, U.S.A.); anti-phosphorylated tau at Ser396 (pS396) (44752G) was purchased from Invitrogen (California, U.S.A.); anti-GSK-3β (Ser9) (#9322) was purchased from Cell Signaling Technology (Beverly, MA, U.S.A.); DAPI was purchased from Roche (Switzerland); anti-tubulin antibody (ab28035) was purchased from Abcam (U.S.A). Cy5-labeled Donkey anti-Rabbit/Donkey anti-Mouse secondary antibodies (711-175-152)/(715-175-150) were purchased from Jackson ImmunoResearch (West Baltimore Pike, PA, U.S.A.).

Chemicals: Dulbecco's Modified Eagle's Medium (DMEM)/F12 was purchased from Gibco (U.S.A.). Fetal bovine serum (FBS) was purchased from Hyclone (U.S.A.). The cell viability assay kit CCK8 was purchased from Dojindo Laboratories (Japan). The Annexin-V/PI cell apoptosis assay kit was purchased from KeyGen Biotech (China). Dimethyl sulfoxide (DMSO) was purchased from Amresco (U.S.A.). Trypsin was purchased from Life Technologies (U.S.A.). RIPA cell lysis buffer, BCA protein assay kit and penicillin-streptomycin solution were purchased from Beyotime (China). PVDF transfer membrane and chemiluminescent HRP substrate were purchased from Millipore (U.S.A.). Protease inhibitor cocktail (G6521) for cell lysates was purchased from Promega (U.S.A.). Methanol-free formaldehyde (16% w/v) was purchased from Thermo Scientific (U.S.A.). Resveratrol standard was purchased from Sigma (St. Louis, MO, U.S.A.) and was first dissolved in 50% DMSO and then diluted to various concentrations with culture medium immediately before use. Other reagents, unless otherwise noted, were of analytical grade and purchased from local distributors.

### Cell culture

The mouse neuroblastoma N2a cell line was purchased from Kunming Institute of Zoology, Chinese Academy of Sciences (Kunming, China). The cells were cultured in Dulbecco's Modified Eagle's Medium (DMEM)/F12 medium supplemented with 10% fetal bovine serum (FBS) and 1% Penicillin-Streptomycin in 100-mm culture dishes. Cells were incubated in a humidified incubator at 37°C with 5% CO_2_.

### Experimental design

N2a cells were divided into the following groups: blank control group (cells without any treatment) and vehicle control group (0.025% DMSO pre-treatment + FA treatment); FA group (FA applied at an appropriate concentration according to each experimental scale); Res group (Res applied at an appropriate concentration according to each experimental scale); and Res + FA group (Res pre-, simultaneous-, and post-treatment were performed according to each experimental scale). Cell viability, morphological changes and apoptosis rates were used to evaluate cytotoxicity. Expression levels of tau protein and candidate enzymes were measured by western blotting and immunofluorescence assays.

**Figure 1:**
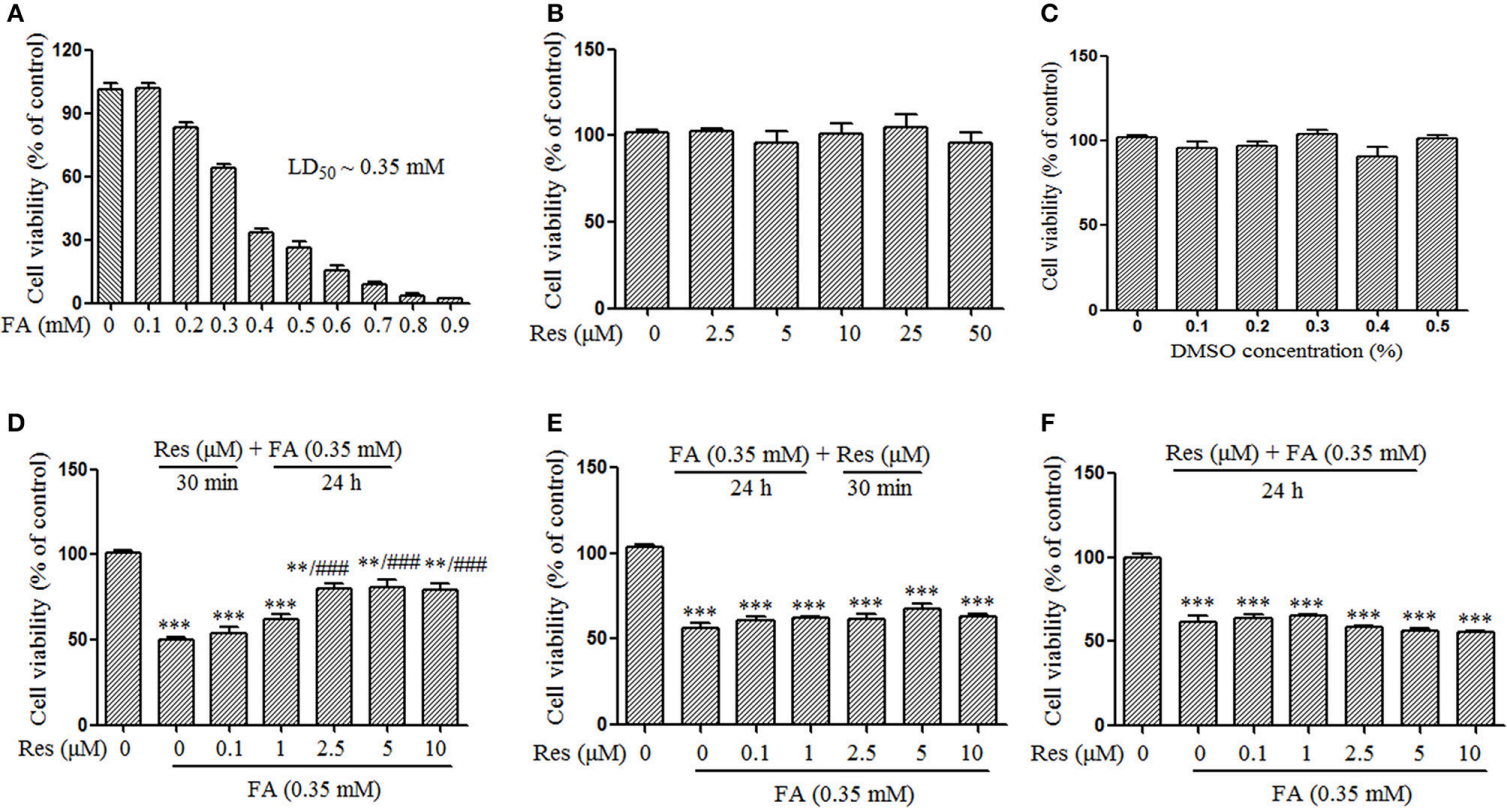
**Protective effect of Resveratrol (Res) against formaldehyde (FA)-induced cytotoxicity in N2a cells, including the respective effect of FA (A)**, Res **(B)**, and DMSO **(C)** on the viability of N2a cells. N2a cells were treated with FA (0.1–0.9 mM), Res (2.5–50 μM), and DMSO (0.1–0.5%) alone for 24 h. Then cell viability was detected with a CCK-8 assay. The protective effect of Res against FA-induced cytotoxicity was investigated in N2a cells. N2a cells were pre-treated **(D)** or post-treated **(E)** with Res (0.1, 1, 2.5, 5, and 10 μM) for 30 min before or after FA exposure for 24 h, or treated simultaneously **(F)** with 0.35 mM FA for 24 h. Cell viability was detected with a CCK-8 assay. All data are presented as the mean ± SEM from independent experiments performed in triplicate (*n* = 3). ^**^p < 0.01, ^***^p < 0.001 compared to control group. ###*p* < 0.001 vs. FA treatment group.

A FA concentration of 0.35 mM was selected to evaluate the timing of Res administration (pre-, simultaneous-, or post-treatments) because it was the LD_50_ of FA in N2a cells found in preliminary studies (Figure 1A). In the western blotting and apoptosis studies, the concentration of FA was increased to 0.5 mM because this FA concentration had been shown to cause marked hyperphosphorylation of Tau protein in N2a cells (Lu et al., 2011; Tong et al., 2011). This increase served to both push out molecular indicators of FA toxicity to evaluate the neuroprotective effect of Res and to evaluate beyond the physiologically defined concentration levels of FA in the brain (0.2–0.4 mM; Chen et al., 2009) in accordance with the upper levels of FA concentrations (~0.5 mM) reported in the hippocampus of AD brains (Tong et al., 2013).

### Measurement of cytotoxicity

Cell viability was determined using the Cell Counting Kit-8 (CCK-8) according to the manufacturer's instruction. Briefly, N2a cells were seeded at 1.5 × 10^4^ cells/well in a 96-well plate (Corning, U.S.A.) and cultured for 24 h to allow cell adhesion. Subsequently, the medium was replaced with FBS-free DMEM/F12 and cells were respectively treated with FA (0.1–0.9 mM), Res (2.5–50 μM), DMSO (0.1–0.5%,) for 24 h. To investigate whether Res could attenuate FA-induced cytotoxicity, cells were also pre- and post-treated with Res (0.1–10 μM) for 30 min before and after 24 h FA (0.35 mM; LD_50_; Figure 1A) exposure as well as treated simultaneously with 0.35 mM FA for 24 h. The optical absorbance at 450 nm was measured on a spectrum plate reader (Synergy H1, BioTek, U.S.A.) and cell viability was expressed as the percentage of viable cells relative to untreated control cells.

To observe cellular morphology changes, cells (1.5 × 10^5^ cells/well) were seeded in a 12-well plate and after a 24 h attachment period the cells were treated with Res (0.1–10 μM) for 30 min followed by a 4 h FA (0.5 mM) exposure. The cells were fixed with 4% paraformaldehyde (PFA) for 10 min and observed with a Fluorescent Cell Imager (ZOE, Bio-Rad, U.S.A.).

Cell apoptosis was detected with an Annexin-V/PI Staining Kit according to the manufacturer's instructions. Briefly, cells (1.5 × 10^5^) were seeded in a 12-well plate, after treatment with Res (0.1–10 μM, 30 min) and FA (0.5 mM, 4 h), cells were digested with 0.25% EDTA-free Typsin. The cells were then collected and washed with cold phosphate-buffered saline (PBS). After washing, the cells were re-suspended in 500 μl of binding buffer containing 5 μl Propidium Iodide (PI) and 5 μl Annexin V-FITC, and incubated for 15 min at room temperature in the dark. Cells were then quantitatively analyzed by Flow cytometer (Influx, BD, U.S.A.). Ten thousand cells were counted one time for each sample. The apoptosis rate was analyzed using the Flowjo 7.6.1 (FlowJo, LLC, U.S.A.) software. The following controls were used to set up compensation and quadrants: (1) unstained cells, (2) cells stained with FITC Annexin V (no PI), and (3) cells stained with PI (no FITC Annexin V). The unstained cells (cells without any treatment) were used to measure the forward scatter (FSC) and side scatter (SSC) signals, which were used to find viable cells and remove signals for debris and dead cells. Cellular debris was considered FSC-low and dead cells were considered to have low FSC and high SSC. A gate was set to exclude debris and dead cells from the analysis by drawing polygons that included the events with high FSC and low SSC (viable cells). Once the gate was set, viable cells with either FITC or PI single staining were measured to generate the FITC positive (vertical axis) or PI positive (horizontal axis) lines, respectively. The boundary of each line was referenced to the unstained viable cells. These two parameters were used to produce a bivariate histogram, which divided the plot into the four quadrants displaying the four possible combinations: double positive (Q2, Annexin V^+^/PI^+^, late apoptotic cells), single positive for each dye (Q1, Annexin V^−^/PI^+^, necrotic or mechanically damaged cells; Q3, Annexin V^+^/PI^−^, early apoptotic cells), and negative for both (Q4, Annexin V^−^/PI^−^, non-apoptotic cells). The four quadrant gates were then applied to the other samples to quantify the percentage of apoptotic cells. The cell apoptotic rate was calculated as Q2+Q3.

### Western blotting assays

For western blotting assays, cells were seeded at 1.5 × 10^5^ cells/well in a 12-well plate and were pre- and post-treated with 10 μM Res for 30 min before and after 4 h FA (0.5 mM) treatment as well as simultaneously treated with 10 μM Res and 0.5 mM FA. Moreover, pre-treatments of 0.1–10 μM Res for 30 min before 4 h FA (0.5 mM) exposure were also performed. Western blotting procedures were performed as described in the literature (Lu et al., 2013) with small modifications. Briefly, whole-cell lysates were prepared by incubation of cells in RIPA buffer supplemented with protease inhibitor cocktail according to the manufacturer's instructions. The protein content was determined using a BCA protein assay kit. Then samples (20 μg of protein) were mixed with loading buffer, heated at 98°C for 10 min, separated on 12% SDS-PAGE gels, and transferred to PVDF membranes. After blocking with 5% skim milk for 1 h, the membranes were incubated with primary antibodies (β -actin, 1:5000; Tau5, 1:2500; pT181, 1:4000; GSK-3β , 1:1000; GSK-3β (Ser-9, phosphor-Serine 9), 1:1000; GSK-3β (Y216, phosphor-tyrosine 216), 1:250; CaMKII, 1:5000; PP2A, 1:1000) at room temperature for 1.5 h or at 4°C overnight. The membranes were then washed three times with TBST for 10 min each, followed by incubation with the corresponding anti-rabbit/anti-mouse IgG horseradish peroxidase (HRP)-conjugated secondary antibodies (1:5000) at room temperature for 1 h. The membranes were again washed three times with TBST (10 min each time) and visualized with chemiluminescent HRP substrate according to the manufacturer's instructions. Luminescence was captured in the ImageQuant LAS 4000 digital imaging system (GE Healthcare Bio-Sciences, Pisacataway, U.S.A.). The individual band intensities were quantified with the Image J software package (National Institute of Health, Besthada, U.S.A.) and normalized to the intensity of β-actin bands. This allowed individual bands to be compared to the total protein on each membrane. The quantification of GSK3β-Y216 band intensity was measured as the total of all bands, as per a previous study of GSK3β post-translational measurements (Beurel et al., 2015). The intensity of the lowest band alone was also quantified.

### Immunofluorescence assays

Immunofluorescence assays were performed to further confirm the presence of phosphorylated tau (pT181) and related enzymes according to a previously established protocol (Wei et al., 2016). In brief, cells were seeded at 1.5 × 10^4^ cells/well on sterile coverslips placed in 24-well culture plates. Cells were pre-treated with 10 μM Res for 30 min before a 0.35 mM FA treatment for 4 h. After treatment, cells were washed three times with PBS (5 min each time) and fixed with 4% PFA for 20 min at 4°C, then permeabilized with 0.4% Triton X-100 for 30 min at room temperature. The coverslips were then blocked with 5% bovine serum albumin (BSA) for 1 h, followed by an incubation in 1% blocking buffer with primary antibodies (tublin, 1:1000, pT181, 1:100; GSK-β, 1:1000; GSK-β (Y216), 5 μg/ml; GSK-3β (Ser9), 1:100; CaMKII, 1:250; PP2A, 1:250; Tau2, 1:500) at 4°C overnight. Cells were washed three times with PBS (5 min each time) to remove residual primary antibodies. Cy5-labeled Donkey anti-Rabbit/Donkey anti-Mouse secondary antibodies (1:200) were added and allowed to bind at 37°C for 1 h in the dark. The coverslips were then washed as mentioned earlier and were incubated with DAPI (1:1000) for 10 min in the dark. Following three 5-min washings with PBS, the slides were mounted with 90% glycerol (Solarbio, China), then sealed with nail polish. Slides were observed under a laser scanning confocal microscope (A1MP+, Nikon, Japan).

### Statistical analysis

Cell viability and western blot data analysis were conducted using the SPSS software package (SPSS Inc., Chicago, IL, U.S.A.). The normality of the data was determined using the Kolmogorov-Smirnov test. All the data were normally distributed (Kolmogorov-Smirnov tests: all *p* > 0.05) and were analyzed by one-way ANOVA. The alpha level was set at *p* = 0.05 and all *p*-values were generated using two-sided tests. Tukey's tests were used for *post-hoc* comparisons. Results were calculated over at least three independent experiments and were presented as the mean ± SEM (standard error of the mean).

## Results

### Neuroprotective effects of Res against FA-induced cytotoxicity

Slow progressive neuronal death is a major characteristic of AD (Shimohama, 2000). In this study, the protective effect of Res against FA-induced neuronal toxicity was studied in N2a cells. FA induced cell death in a dose-dependent manner with an LD_50_ of ~0.35 mM (Figure 1A). Data from the CCK-8 assays showed that Res alone (2.5–50 μM) did not cause any loss of N2a cell viability after 24 h treatments (Figure 1B). Similar results were observed when N2a cells were treated with different concentrations of DMSO (0.1–0.5%), the vehicle used for Res (Figure 1C). To determine whether Res attenuates FA-induced cytotoxicity, cell viability assays were performed with pre- and post-treatments of Res (0.1–10 μM) for 30 min, as well as with simultaneous-treatments of Res, with FA for 24 h. Pre-treatment with Res dose-dependently attenuated FA-induced cytotoxicity (Figure 1D), with a significant effect observed at a concentration of 2.5 μM (*p* = 6.728 × 10^−5^). The viability of N2a cells was not influenced by post-treatments (Figure 1E, *p* = 0.497) or simultaneous-treatments (Figure 1F, *p* = 0.371) of Res.

**Figure 2:**
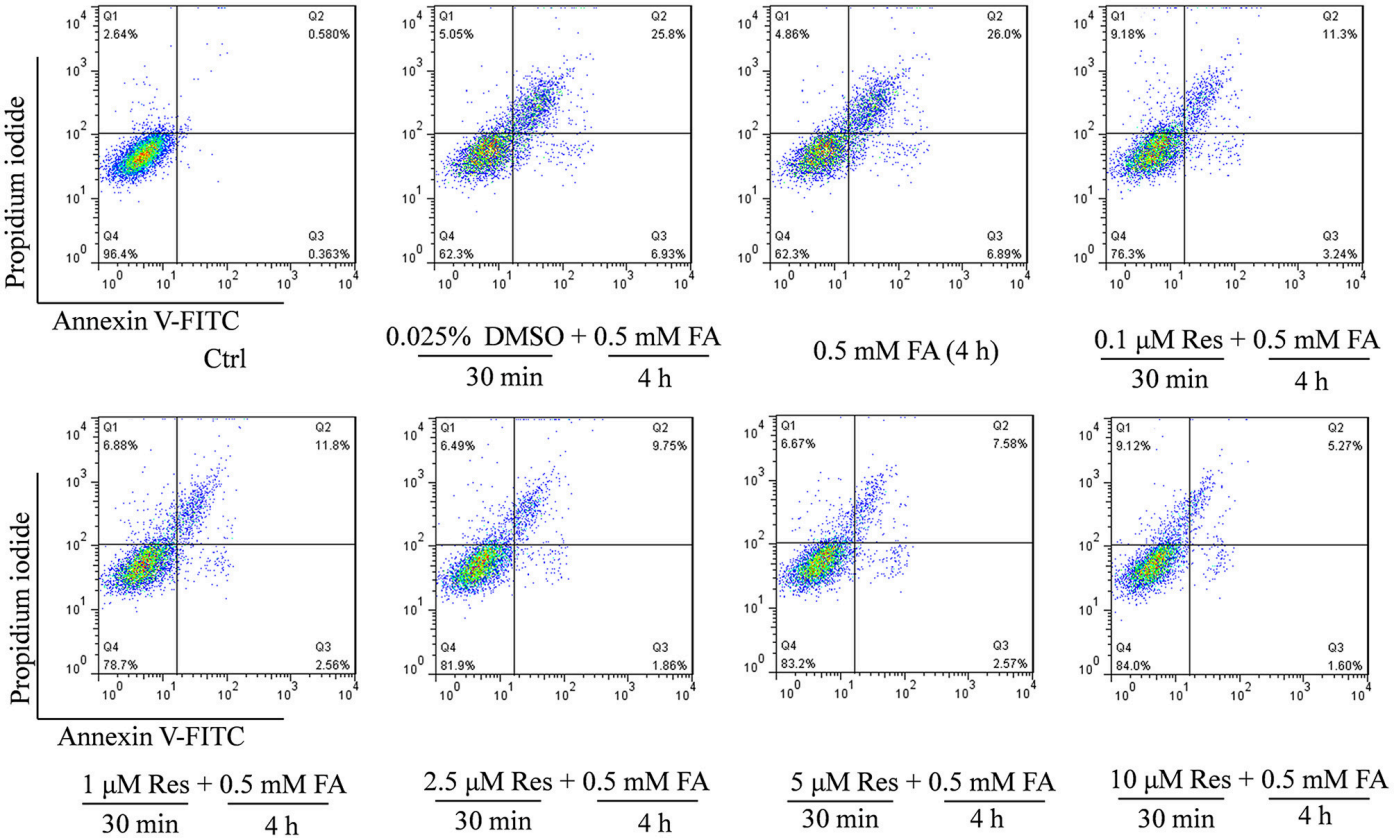
**Protective effect of Res against FA-induced cell apoptosis**. N2a cells were pre-treated with Res (0.1, 1, 2.5, 5, and 10 μM) for 30 min and then treated with 0.5 mM FA for 4 h. Cell apoptosis rates were measured with Annexin-V/PI double staining. A single apoptotic assay was performed with 10,000 cells quantitatively analyzed per group in a Flow cytometer. The Annexin V-FITC vs. Propidium Iodide (PI) gates denote the following populations: necrotic or mechanically damaged cells (Q1, Annexin V^−^/PI^+^), late apoptotic cells (Q2, Annexin V^+^/PI^+^); early apoptotic cells (Q3, Annexin V^+^/PI^−^) and non-apoptotic cells (Q4, Annexin V^−^/PI^−^). The cell apoptotic rate was calculated as Q2+Q3.

In addition, an Annexin V-FITC/PI double staining assay was used to briefly evaluate the respective effect of FA and Res on N2a cell apoptosis. The experiments found that 0.5 mM FA increased the apoptotic rate to 32.7% in comparison to the control group (0.9%) (Figure 2). However, pre-treatment with Res (0.1, 1, 2.5, 5, and 10 μM) given prior to FA (0.5 mM) exposure decreased the percentage of apoptotic cells to 14.5, 14.4, 11.6, 10.2, and 6.9%, respectively (Figure 2). These results were consistent with the findings from the cell viability assays, and, together, showed that pre-treatment of Res protected N2a cells against FA-induced cytotoxicity.

**Figure 3:**
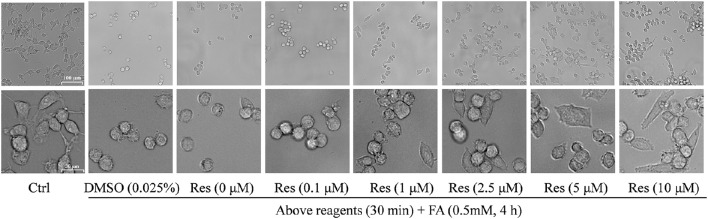
**Protective effect of Res against FA-induced cell morphological changes**. N2a cells were pre-treated with Res (0.1, 1, 2.5, 5, and 10 μM) for 30 min, and then treated with 0.35 mM FA for 4 h. Cell morphology was observed using a microscope at different magnifications (upper bar: 100 μm; lower bar: 30 μm). The images show morphological changes of cells with or without Res treatment.

Cellular morphology was also observed using a microscope at different magnifications. Compared with controls, FA-treated cells were smaller and atrophied with irregular cell borders and reduced cellular processes, while cells pre-treated with Res showed a higher cell density and a more natural cell morphology. Moreover, cell neurites were partially retained in comparison with cells treated with FA alone (Figure 3).

In consideration of the functions of the tau protein to promote the assemble of microtubules and maintenance of the microtubule system in neurons (Drechsel et al., 1992; Götz et al., 2006), as well as findings that abnormal phosphorylation of tau protein leads to microtubule disassembly, cytoskeleton destabilization and eventually cell death (Iqbal et al., 2005), it was hypothesized that the protective effect of Res against FA-induced neurotoxicity and morphological changes was likely related to the modulation of tau protein phosphorylation.

### Effects of Res on FA-induced tau protein hyperphosphorylation

**Figure 4:**
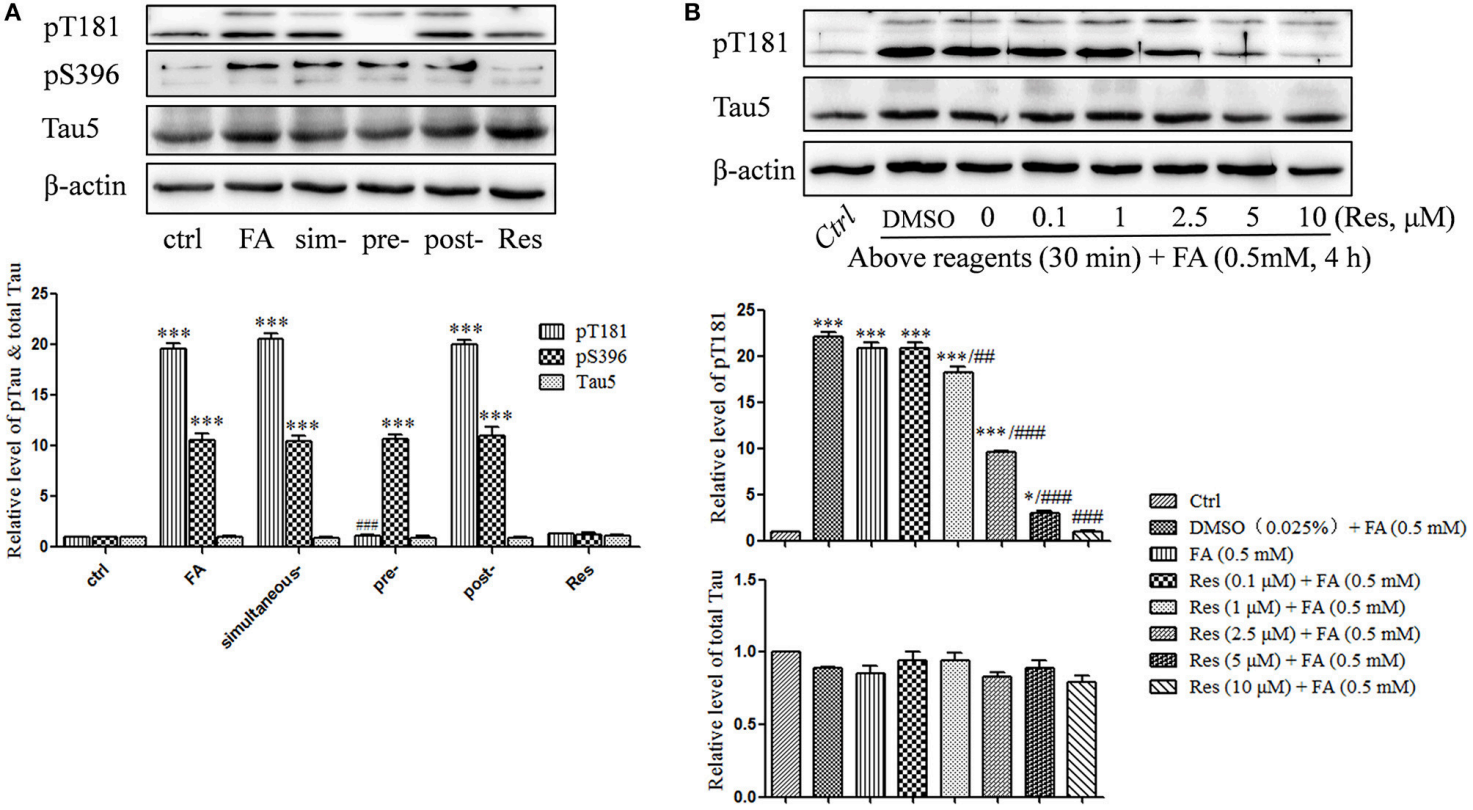
**Res attenuates FA-induced tau protein hyperphosphorylation in N2a cells. (A)** N2a cells were pre-treated or post-treated with 10 μM of Res for 30 min before or after 0.5 mM FA exposure for 4 h, or treated simultaneously with 0.5 mM FA for 4 h. **(B)** N2a cells were pre-treated with Res (0.1, 1, 2.5, 5, and 10 μM) for 30 min, and then treated with 0.5 mM FA for 4 h. Total and phosphorylated tau protein levels were determined by Western blotting **(A,B)**. Densitometry values were calculated from independent experiments performed in triplicate (*n* = 3) and presented as mean ± SEM. ^*^p < 0.05, ^***^p < 0.001 vs. control. ##*p* < 0.01, ###*p* < 0.001 vs. FA treatment group.

Previous studies indicated that FA triggered abnormal tau protein hyperphosphorylation *in vitro* and *in vivo* (Nie et al., 2007; Lu et al., 2013). In this study, tau protein hyperphosphorylation at Thr181 (*p* = 6.965 × 10^−9^) and Ser396 (*p* = 2.750 × 10^−7^) were significantly increased after treatment with 0.5 mM FA (Figure 4A). Pre-treatment with 10 μM Res significantly reduced FA-induced tau phosphorylation at Thr181 (*p* = 7.456 × 10^−9^), but not at the Ser396 site, which did not change significantly (*p* = 1.000). As in the cell viability and apoptosis studies above, post- (*p* = 0.930 for Thr181 and *p* = 0.991 for Ser396) or simultaneous-treatment (*p* = 0.994 for Thr181 and *p* = 1.000 for Ser396) of 10 μM Res did not influence tau phosphorylation levels at either the Thr181 or Ser396 loci (Figure 4A). It was further elucidated that pre-treatment with 0.1–10 μM Res reduced FA-induced tau hyperphosphorylation at Thr181 in a dose-dependent manner (*p* = 0.007 at 1 μM of Res; Figure 4B). The expression level of total tau protein did not show a significant change with (*p* = 0.979 at 10 μM of Res) or without (*p* = 0.321) Res treatment (Figure 4B), which indicated an overall net effect of Res that suppressed FA induced tau protein hyperphosphorylation. All told, these results suggested that Res significantly inhibited tau protein hyperphosphorylation at Thr181, but not at Ser396, in a dose-dependent manner.

### Effects of Res on kinase (GSK3β/CaMKII) inactivation and phosphatase (PP2A) activation

**Figure 5:**
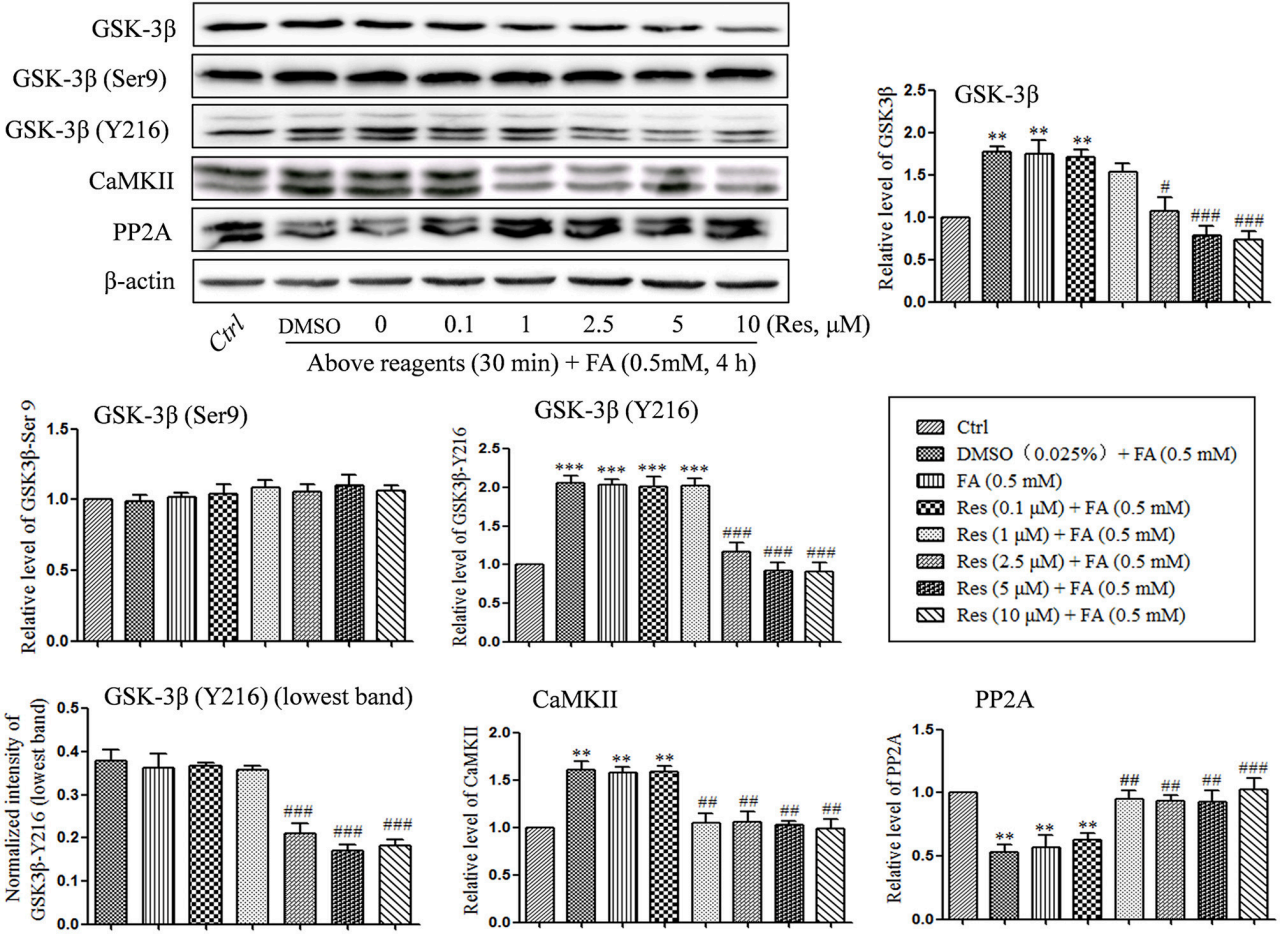
**Res down-regulated the level of GSK-3β and CaMKII and up-regulated the level of PP2A**. N2a cells were pre-treated with Res (0.1, 1, 2.5, 5, and 10 μM) for 30 min, and then treated with FA (0.5 mM) for 4 h. Cells were collected and the protein levels of GSK-3β, CaMKII and PP2A were detected by Western blot analysis. Densitometry values were calculated from independent experiments performed in triplicate (*n* = 3) and presented as mean ± SEM. ^**^p < 0.01, ^***^p < 0.001 vs. control. #*p* < 0.05, ##*p* < 0.01, ###*p* < 0.001 vs. FA treatment group.

Kinases and phosphatases, such as glycogen synthase kinase (GSK-3β), calmodullin-dependent protein kinase II (CaMKII) and phosphoseryl/ phosphothreonyl protein phosphatase-2A (PP2A), are important enzymes involved in the regulation of hyperphosphorylation of tau protein in the development of AD (Gong et al., 1993, 1995; Pei et al., 1999; Vogelsberg-Ragaglia et al., 2001; Wang et al., 2005). In the present study, the effects of Res on the regulation of GSK-3β, CaMKII, and PP2A activities were investigated. It was found that the level of phosphorylation on GSK-3β at Y216 (activation site) was significantly increased after treatment with 0.5 mM FA for 4 h (*p* = 1.974 × 10^−5^; Figure 5), while phosphorylation at Ser9 (inhibition site) did not exhibit a significant change (*p* = 1.000). This suggested an increased activity of total GSK-3β (*p* = 0.005). Conversely, pre-treatment with Res significantly decreased the level of phosphorylation on GSK-3β at Y216 (*p* = 1.722 × 10^−4^ at 2.5 μM of Res). This indicated that the phosphorylation of GSK-3β at the activation site was inhibited by Res treatment (Figure 5). However, phosphorylation of GSK-3β at the Ser9 site was not found to change significantly with Res pre-treatment prior to FA exposure (Figure 5, *p* = 0.997 at 10 μM of Res).

An analogous result was found with CaMKII. The protein level of CaMKII was significantly increased under FA stress (*p* = 0.002), while pre-treatment with Res significantly reduced the level of CaMKII in a dose-dependent manner (Figure 5, *p* = 0.005 at 1 μM of Res).

With respect to the phosphatase PP2A, a significantly decrease in the protein level of PP2A was found after FA exposure (*p* = 0.008), while pre-treatment with Res significantly increased the protein levels (Figure 5, *p* = 0.020 at 1 μM of Res). These results suggest that Res protects N2a cells from FA-induced tau protein hyperphosphorylation by decreasing the activities of GSK-3β and CaMKII as well as increasing the activity of PP2A. Interestingly, no significant changes on the regulation of GSK-3β, CaMKII and PP2A activities and/or expression were found after 30 min of Res treatment alone (data not shown).

**Figure 6:**
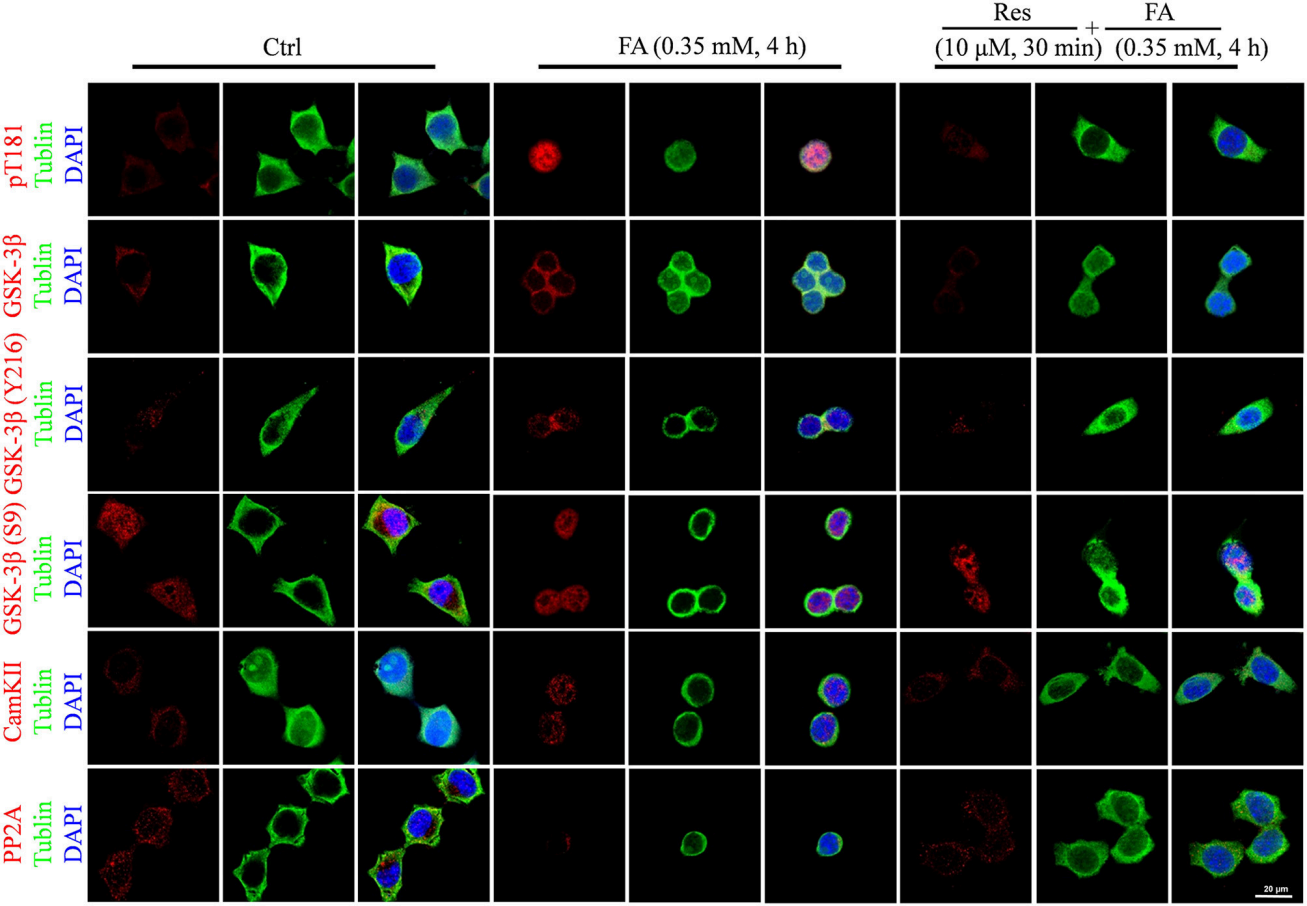
**Immunofluorescence staining of N2a cells labeled for phosphorylated tau protein (Thr181), GSK-3β (total and Ser9/Y216 site), CaMKII, and PP2A (Bar: 20 μm)**. N2a cells were pre-treated with 10 μM Res for 30 min and then treated with FA (0.5 mM) for 4 h. Fixed cells immunostained as labeled were observed for changes in the fluorescence of phosphorylated tau (Thr181, red), GSK-3β (total and Ser9/Y216 site, red), CaMKII and PP2A (red), DNA (DAPI, blue), and cytoskeleton (Tubulin, green) by fluorescence confocal microscopy. Each test was repeated three times and 10 cells/coverslip were imaged for each repeat.

Alterations to tau protein hyperphosphorylation, kinase, and phosphatase levels and protein localizations were further investigated by immunofluorescence staining. An increased staining signal of tau (pT181), GSK-3β, and CaMKII as well as a decreased staining pattern of PP2A were detected after FA treatment (Figure 6). These staining patterns were also ameliorated with pre-treatments of Res and were consistent with the findings of the Western blot analysis.

## Discussion

In this study, FA exposure was observed to induce AD markers *in vitro*, including tau protein hyperphosphorylation and neuronal death. Pre-treatment with Res was found to significantly decrease FA-induced cytotoxicity, to reduce cellular apoptosis rates and to inhibit tau protein hyperphosphorylation. It was further revealed that Res protected the cells from FA-induced damage by modulating the activities of GSK-3β, CaMKII kinase, and PP2A phosphatase.

As AD is a global health problem that is accelerating its reach with an increasingly aging population (Gong, 2014), it has become urgent to develop new therapies for successful prevention and management of this chronic debilitating disease. In recent studies, it has been implicated that FA is one of the factors involved in the initiation and progression of AD (Kilburn et al., 1987; Nie et al., 2007; Tong et al., 2011, 2013; Lu et al., 2013; Rizak et al., 2014), which suggests that new therapies may be developed for the treatment of AD by protecting neurons against FA-induced damages.

Res, a polyphenol anti-oxidant, was observed in this study to protect N2a cells against FA-induced cytotoxicity and tau protein hyperphosphorylation. It was further identified that the neuroprotective effect of Res was related to a signaling cascade which modulated the activities of kinases and a phosphatase involved in tau protein phosphorylation. FA was also found to induce cell morphological changes and cell apoptosis in this and other studies (Lu et al., 2013). In the present study, pre-treatment with Res was demonstrated to protect N2a cells against FA-induced cytotoxicity by increasing cell viability, decreasing cell apoptotic rates and maintaining cell morphology. This protective effect was found to occur in a dose-dependent manner with significant effects observed at concentrations of 2.5 μM or greater. Although this concentration was 140 times lower than that of FA (0.35 mM), the pre-treatment of Res was sufficient to protect the N2a cells from FA-induced damages. Simultaneous- or post-treatments of Res did not protect or improve damage to the cells, which is in accordance with the findings from a previous study where simultaneous treatments of equal doses of Res and FA (such as 100 μM) did not reverse FA-induced cytotoxicity (Marcsek et al., 2007). In the case of this study, the concentration of FA was much higher than Res, which suggested the ability of Res to capture FA directly may be overshadowed. The results and concentrations in this study suggested that the protective effect of Res against FA-induced cytotoxicity was mediated through a signaling cascade, rather than the direct interaction of Res, as a natural formaldehyde capturer (Tyihák et al., 1998), with FA.

It was then hypothesized that Res exerted this protective effect by regulating the phosphorylation state of tau protein because abnormal tau protein hyperphosphorylation can result in the disassembly and depolymerization of microtubules leading to cell death (Obulesu et al., 2011; Duan et al., 2012). In this study, a significantly increased tau protein hyperphosphorylation at Thr181 and Ser396 was observed after FA treatment, which was consistent with previous studies (Nie et al., 2007; Lu et al., 2013). Pre-treatment with Res significantly inhibited FA-induced tau phosphorylation at Thr181 at a relatively low dosage (2.5 μM). This suggests that Res protects N2a cells against FA-induced neurotoxicity through inhibiting tau protein hyperphosphorylation. Interestingly, pre-treatment of Res influenced FA-induced tau phosphorylation at Thr181 but not at Ser396, which suggested that this protective effect was site-specific.

The hyperphosphorylation of tau protein is regulated by phosphorylation of a number of kinases (such as GSK-3β and CaMKII) and the dephosphorylation activity of a phosphatase (PP2A) (Wang et al., 2007). To further elucidate the role of Res in reducing FA-induced tau protein hyperphosphorlation, the protein levels of these enzymes and the phosphorylation state of GSK-3β were investigated in this study. It was found that the phosphorylation of GSK-3β at Y216 site (activated form) was increased after FA treatment, while the phosphorylation at Ser9 site (inactivated form) did not change significantly. This suggests that GSK-3β activation contributed to the hyperphosphorylation of tau protein, which is similar to a previous study that demonstrated FA triggered tau protein hyperphosphorylation via GSK-3β signaling (Lu et al., 2013).

This study also demonstrated that the level of CaMKII was elevated after FA exposure. CaMKII is considered a tau protein kinase that can augment the subsequent phosphorylation of tau catalyzed by GSK-3β (Wang et al., 1998). Furthermore, FA toxicity induced a decrease in the protein level of PP2A, which has been found to dephosphorylate most abnormal phosphorylation sites on tau protein (Ono et al., 1995; Wang et al., 1995, 1996). These results suggested that the neurotoxic effects of FA were caused, at least in part, by the increased activities of GSK-3β and CaMKII and decreased activity of PP2A. RNA interference experiments with GSK-3β specific siRNA alone did not reduce tau phosphorylation to constitutive levels after FA administration (Lu et al., 2013), further suggesting that FA imparts its toxicity through a number of signaling mechanisms.

In order to determine whether the inhibitory effect of Res on tau protein hyperphosphorylation was due to the modulation of these enzymes, the protein levels of the candidate enzymes mentioned above were investigated following pre-treatment with Res. Consistent with the suppressive effect of Res on tau protein hyperphosphorylation, pre-treatment with Res significantly inhibited FA-induced increasing phosphorylation of GSK-3β and CaMKII protein level. This indicated that regulation of these two kinases is important in the attenuation of FA-induced hyperphosphorylation of tau protein by Res. In addition, it was found that pre-treatment of Res significantly increased the protein level of PP2A where FA exposure was found to down-regulate PP2A levels. This suggests that PP2A may also be involved in the neuroprotective effect of Res against tau protein hyperphosphorylation. All these results indicate that Res inhibited tau protein hyperphosphorylation by regulating the activities of GSK-3β, CaMKII and PP2A, all of which are important enzymes known to modulate the homeostasis of tau protein phosphorylation (Wang et al., 2007).

Interestingly, Res treatments alone were not found to modulate the baseline levels of the enzymes GSK-3β, CaMKII, and PP2A in the 30 min prior to the addition of FA (data not shown). This is a complicated result that suggests that the neuroprotective effect of Res against FA toxicity either works upstream through an unknown mechanism to prime the cells against toxic insults or that the effects of Res manifest after the 30 min incubation period. Further investigation into the up-stream modulation of these signaling pathways, both in relation to toxic insult and Res neuroprotection, may uncover new knowledge applicable to the treatment of Alzheimer's disease.

In summary, the present study provided the first evidence that Res exerts a significant neuroprotective effect on N2a cells against FA-induced damages through the inhibition of tau protein hyperphosphorylation. The molecular mechanisms behind this inhibition is likely related to the modulation of GSK-3β, CaMKII and PP2A activities. Taken together, this study extended our understanding of the neuroprotective effects of Res and suggested that Res modulates a number of enzymes specifically involved in the regulation of tau protein hyperphosphorylation, which is considered an important drug target in the treatment of AD pathology.

## Author contributions

XPH and ZL designed and carried out the experiments; JR provided scientific discussion and revised the manuscript; XTH advised on data presentation and provided financial support; JW advised on data presentation and provided unofficial financial support. SW, ZW, RH, MS, and DQ participated in analyzing and interpretation of the results; all authors were involved in writing, reading and final approval of the submitted manuscript.

## Funding

This research was supported by the National Program for Key Basic Research Projects (973 Programs 2015CB755605, 2012CB825503, 2012CBA01304), the Strategic Priority Research Program of the CAS (XDB02020005), the Key Research Program of the Chinese Academy of Sciences, the Selected Frontier Scientific Significant Breakthrough Project of the CAS, the Key Program of the Chinese Academy of Sciences (KZCC-EW-103-2), the Training Program of the Major Research Plan of the National Natural Science Foundation of China (91332120), the National Natural Science Foundation of China (81471312, 31271167, 81271495, 81460352), the Yunnan Provincial Project to Attract One-hundred Exceptional Talents From Overseas and the Applied Basic Research Programs of Science and Technology Commission Foundation of Yunnan Province (2014FA047).

### Conflict of interest statement

The authors declare that the research was conducted in the absence of any commercial or financial relationships that could be construed as a potential conflict of interest. The reviewer RJR and handling Editor declared their shared affiliation, and the handling Editor states that the process nevertheless met the standards of a fair and objective review.
